# Flavor Formation and Quality Maintenance in Meat Processing

**DOI:** 10.3390/foods12193678

**Published:** 2023-10-07

**Authors:** Ying Wang, Yani Luo, Jinxuan Cao

**Affiliations:** School of Food and Health, Beijing Technology and Business University, Beijing 100048, China; wangying4@nbu.edu.cn (Y.W.); luo_yny@sina.com (Y.L.)

As an important source of nutrients, meat can supply protein, fat, vitamins and minerals, which are crucial in people’s diet worldwide. A rapid development of the global economy, population and industrialization has inevitably aroused the exploitation of the meat industry, which has eventually fueled the market for meat products. In recent decades, the meat industry has witnessed enormous changes in different geographical regions. Global meat consumption has increased notably since the 1960s and witnessed a 500% rise from 1992 to 2016 [1]. Tremendous increase in meat consumption has happened especially in Asia, Latin America and Africa. Almost half of the global pork production and consumption occurs in China and a few other countries in Southeast Asia [2]. The per capita consumption of meat is influenced strongly by consumer preferences and income growth.

The diversity of food products supplies more choices for consumers. Under different sociocultural contexts, consumers’ perception of meat products, which plays a crucial role in the profitability of the meat industry, is becoming more complex [3]. Consumers have individual preferences for flavors and perceived quality of meat products. The wide-ranging demands encompass not only the sensory quality of meat products, but also the price, convenience, safety, nutrition and texture. These expectations place increasing pressure on the industry and boost the search for new meat processing strategies, which impact the taste, aroma, texture and overall consumer satisfaction of meat products. 

Meat flavor, which involves the stimulation of taste buds mostly by volatile compounds, is a crucial sensory property characteristic of overall acceptability. Raw meat usually has an unpleasant flavor, such as a metallic, salty and bloody taste, and an off-flavor. The flavor of raw meat is influenced by the diet, breed, age and intramuscular fat of animals. Upon processing, cooked meat usually possesses a unique and attractive flavor with rich aromas that are ascribed to a series of complex chemical reactions, such as lipid oxidation, Maillard reaction, protein oxidation and protein–phenol reactions [4]. Through the appropriate regulation of conditions in meat processing, the flavor of meat can be monitored. In addition, ingredients are usually utilized to enhance the flavor of meat. Examples include seasonings, spices and additives. 

The quality of meat refers to the overall characteristics, such as appearance, texture, flavor, tenderness, juiciness, nutritional value and safety. It is worth noting that meat quality can vary depending on the processing methods, packaging and storage. 

To enhance the flavor and quality of meat, various processing methods have been developed from ancient times to the present. Freezing is usually used to preserve raw meat and meat products through slowing down the rate of chemical reactions. It has been reported that well-preserved frozen meat has a storage period of more than two years with the maintenance of eating quality [5]. Steaming, boiling, baking, frying and roasting, which are classified as thermal processing methods, are common methods of processing meat with significant influences on the flavor and quality of meat. The appropriate combination of temperature and time determines the physicochemical characteristics of meat products. Marinating, drying, smoking and fermentation have been used as meat processing techniques worldwide for thousands of years, which not only preserve meat but also impart unique flavors. 

Currently, consumers prefer healthy lifestyles and, thus, seek out natural, fresh and tasty meat products with a low salt content that are free from synthetic additives and preservatives. The complex demands of consumers and the rapid development of food industrialization promote the evolution of the meat processing industry and the improvement of ingredients. A series of processing technologies have been explored in meat processing. Chemical substances, such as chlorine, organic acids and ozone, are usually used in the chemical processing of meat, which exhibit antimicrobial activity, stabilize color, regulate acidity and develop characteristic flavor [6]. High pressure, nanotechnology, irradiation, power ultrasound and microwave are increasingly utilized to improve the flavor and maximize the shelf life of meat products. In recent years, there have been several innovative developments in the fermentation of meat, such as controlled fermentation environments, tailored starter cultures, novel ingredient and flavor combinations, and bioengineering and microbial manipulation.

There are more than 2000 compounds of synthesized food additives in the world, which are important determinants of flavor and food preservation [7]. However, the abuse of additives, including their excessive use or even the use of toxic additives, brings potential risks to the health of people and threatens the trust of consumers in the processing of meat. To date, additive technologies are highly demanded to replace synthetic additives with natural sources without health hazards and quality deterioration. Some starter cultures, spices, essential oils and other natural antioxidants have been found to provide nutritional value, sensory properties and antibacterial ability [8].

This Special Issue aims to publish quality articles about flavor formation and quality maintenance in meat processing, including processing technologies, ingredients and mechanisms.
